# Assessment of Thrombotic Risk in Patients with Tuberculosis and SARS-CoV-2 Coinfection: A Retrospective Study

**DOI:** 10.3390/diagnostics16050724

**Published:** 2026-02-28

**Authors:** Sofia Teodora Muntean, Andreea-Raluca Cozac-Szoke, Diana Maria Chiorean, Adrian Horațiu Sabău, Iuliu Gabriel Cocuz, Raluca Niculescu, Claudia Raluca Mariean, Ovidiu Simion Cotoi, Anca Ileana Sin

**Affiliations:** 1 Doctoral School of Medicine and Pharmacy, George Emil Palade University of Medicine, Pharmacy, Science, and Technology of Targu Mures, 540139 Targu Mures, Romania; sofia.harsan@umfst.ro (S.T.M.); raluca.niculescu@umfst.ro (R.N.); raluca.mariean@umfst.ro (C.R.M.); 2 Department of Pathophysiology, George Emil Palade University of Medicine, Pharmacy, Science, and Technology of Targu Mures, 540139 Targu Mures, Romania; diana.chiorean@umfst.ro (D.M.C.); adrian-horatiu.sabau@umfst.ro (A.H.S.); iuliu.cocuz@umfst.ro (I.G.C.); ovidiu.cotoi@umfst.ro (O.S.C.); 3 Pneumology Department, Clinical County Hospital of Mures, 540142 Targu Mures, Romania; 4 Pathology Department, Clinical County Hospital of Mures, 540011 Targu Mures, Romania; 5 Radiology Department, Emergency Clinical County Hospital of Mures, 540136 Targu Mures, Romania; 6 Department of Genetics and Cellular and Molecular Biology, George Emil Palade University of Medicine, Pharmacy, Science, and Technology of Targu Mures, 540139 Targu Mures, Romania; ileana.sin@umfst.ro

**Keywords:** tuberculosis, COVID-19, SARS-CoV-2, thrombotic risk, Padua score, IMPROVE-DD score, coagulopathy, venous thromboembolism

## Abstract

**Background/Objectives:** Tuberculosis and COVID-19 are two major infectious diseases with significant inflammatory and immunological impact on infected hosts and both conditions are independently associated with a prothrombotic state. However, evidence regarding their combined effect on in-hospital thrombotic risk remains limited. In this study, we aimed to explore whether patients with tuberculosis and COVID-19 coinfection are at a higher risk of developing thrombotic events during hospitalization than patients diagnosed with tuberculosis alone. **Materials and Methods:** We performed a retrospective, single-center cohort study, including adults hospitalized at the Pulmonology Clinic, Adult Tuberculosis ward of Mures County Clinical Hospital, between 2021 and 2023. Two groups were analyzed: patients with pulmonary tuberculosis who developed COVID-19 during hospitalization (*n* = 40) and patients with pulmonary tuberculosis without documented SARS-CoV-2 infection (*n* = 40). Demographic, clinical, laboratory, and imaging data were extracted from medical records. Padua and IMPROVE-DD scores were calculated retrospectively, a rapid mini-score was evaluated exploratorily. Comparisons between groups were performed using appropriate statistical tests and unadjusted odds ratios (ORs) with 95% confidence intervals (CIs) were reported. Given the limited number of events, an age-adjusted Firth penalized logistic regression model was used for multivariable analysis. **Results:** Thrombotic events occurred more frequently in the tuberculosis and COVID-19 co-infection group (22.5% vs. 10%), although statistical significance was not reached (*p* = 0.22; OR = 2.61). Patients with coinfection had significantly higher proportions of elevated Padua scores (55% vs. 20%, *p* = 0.002; OR = 4.88), while IMPROVE-DD showed values near the conventional threshold for statistical significance (37.5% vs. 17.5%, *p* = 0.07). D-dimer values did not reach statistical significance (*p* = 0.07) and platelet counts were significantly higher in patients with tuberculosis only (*p* = 0.001). Mortality did not differ significantly between groups (15% vs. 10%, *p* = 0.73). In age-adjusted multivariable analysis, tuberculosis and COVID-19 coinfection remained associated with higher odds of thrombotic events, with wide confidence intervals. **Conclusions:** Patients with concomitant tuberculosis and COVID-19 showed a higher thrombotic risk profile (Padua score) and numerically higher rates of in-hospital thrombotic events, without reaching statistical significance. Findings should be interpreted as exploratory and hypothesis-generating. Larger prospective studies with systematic imaging and multivariable adjustment are needed.

## 1. Introduction

Tuberculosis (TB) remains a major global infectious disease burden, particularly in low- and middle-income countries, where it continues to contribute substantially to morbidity and mortality [1]. This disease even exists in many high-income countries, despite the availability of preventive vaccination. Despite advances in diagnosis and treatment, TB is frequently associated with chronic systemic inflammation, prolonged hospitalization and multiple comorbidities, all of which may predispose patients to thrombotic complications [2].

Since 2020, this global health problem has been superimposed by the SARS-CoV-2 virus pandemic, which further complicated the management of patients with TB. SARS-CoV-2 infection is known to induce a distinct prothrombotic state, driven by acute systemic inflammation, endothelial dysfunction, platelet activation, and coagulation pathway dysregulation [3]. Both TB and COVID-19 have been independently associated with an increased risk of venous thromboembolism, yet data regarding their combined effect on thrombotic risk remain limited [4].

Patients hospitalized for TB represent a particularly vulnerable population, as SARS-CoV-2 infection may occur during hospitalization and overlap with an already established inflammatory and hypercoagulable milieu [5]. In this context, distinguishing whether TB and COVID-19 coinfection is associated with differences in thrombotic risk profiles or in-hospital thrombotic events compared with TB alone is clinically relevant. These findings are especially important in TB-endemic settings, where resources for systematic imaging and monitoring may be limited.

Current evidence addressing thrombotic risk in TB-COVID-19 coinfection is scarce and largely derived from heterogeneous cohorts, case series, or studies focused primarily on mortality and respiratory outcomes rather than thrombotic complications specifically [6,7,8]. Few studies have explored thrombotic risk stratification using validated prediction tools or have examined thrombotic events specifically in patients hospitalized for active TB who subsequently develop SARS-CoV-2 infection.

Therefore, the present study aimed to explore differences in thrombotic risk profiles and the occurrence of in-hospital thrombotic events between patients with microbiologically confirmed pulmonary tuberculosis who developed SARS-CoV-2 infection during hospitalization and patients hospitalized with pulmonary tuberculosis alone. Given the retrospective design and limited sample size, this analysis was conducted as an exploratory, hypothesis-generating study, with cautious interpretation of findings and explicit acknowledgment of methodological limitations.

## 2. Background and Biological Rationale

In a study conducted in 2015, globally, an estimated 10.2 million incident cases of tuberculosis, 10.1 million prevalent cases and 1.3 million deaths were estimated, of which 1.1 million occurred in HIV-negative people. Age-standardized incidence and mortality were almost twice as high in men as in women, and in children under 15 years of age, and about 690,000 new cases and 70,000 deaths were recorded [1].

The pandemic, according to the World Health Organization, has caused over 776.8 million confirmed cases of COVID-19 and over 7 million deaths worldwide, illustrating a significant morbidity and mortality accumulated during the pandemic [9]. A systematic review [10] published during the COVID-19 pandemic shows that patients infected with the SARS-CoV-2 virus who also had tuberculosis were more than twice as likely to develop severe forms of the disease compared to those without tuberculosis. This study suggests a significantly negative impact of this comorbidity on morbidity. However, mortality did not differ statistically significantly between the two groups, indicating that the association between tuberculosis and COVID-19 mainly influences the clinical course and severity, rather than the ultimate risk of death.

Concomitant infection with tuberculosis and COVID-19 creates a complex biological and clinical context, in which systemic inflammation, immune compromise, and cumulative lung injury can worsen the course of both diseases. Co-infected patients are at higher risk of severe respiratory failure, diagnostic and therapeutic delays and at a public health level. This overlap amplifies the global burden, requiring integrated screening, treatment, and monitoring strategies [5].

A meta-analysis, that included over 60,000 patients, identified an incidence of approximately 14.7% for both venous and arterial thromboembolism in patients infected with the SARS-CoV-2 virus, with an incidence of venous thromboembolism more than 2.5 times higher among critically ill COVID-19 patients admitted to ICUs. The presence of thrombotic events among these patients was associated with a 4.4-fold higher mortality in patients who were not hospitalized and 1.6-fold higher mortality in those who were hospitalized [3].

Another meta-analysis, published by Danwang C. et al. [11], synthesized the available global data on the association between active tuberculosis and the risk of thromboembolic events, including nine observational studies totaling 16,190 patients. The results showed an overall prevalence of venous thromboembolism (VTE) of 3.5% (95% CI: 2.2–5.2), with a prevalence of 5.8% (95% CI: 2.2–10.7) for pulmonary embolism and 1.3% (95% CI: 0.8–2.0) for deep vein thrombosis. The comparative analysis demonstrated that patients with active tuberculosis have an almost threefold higher risk of developing a thromboembolic event (OR 2.90; 95% CI: 2.30–3.67) compared to populations without tuberculosis. The risk is also increased for deep vein thrombosis (OR 1.56) and pulmonary embolism (OR 3.58). These data confirm that venous thromboembolism is not a rare event in patients with active tuberculosis. It highlights the need to implement risk-adapted prevention and anticoagulant prophylaxis strategies. At the same time, the results indicate that systemic inflammation induced by tuberculosis infection and specific treatment (especially the use of rifampicin) may contribute to hemostatic imbalance and increased predisposition to thrombotic events.

SARS-CoV-2 infection is not limited to the respiratory system, but is frequently associated with a systemic hemostatic disorder, known as COVID-19-associated coagulopathy. This clinical entity is characterized by a spectrum of thrombotic manifestations, including venous thromboembolism (VTE), especially deep vein thrombosis (DVT) and pulmonary embolism (PE), as well as arterial thrombotic events [12].

The pathophysiological mechanisms involve excessive activation of the innate immune system and the complement system, leading to a cytokine storm, platelet activation and aggregation, endothelial dysfunction. These are all mediated by virus binding to the angiotensin-converting enzyme 2 (ACE2) receptor and impaired fibrinolysis. These processes lead to a persistent procoagulant state and the formation of diffuse microthrombi in the pulmonary and systemic circulation [13]. In severe forms of the disease, the incidence of VTE is higher than in milder forms, which explains the increased mortality rate. Multicenter studies from the United States and Europe have reported high prevalences of VTE in critically ill COVID-19, supporting the need for careful consideration of anticoagulant prophylaxis [14].

The study published by Toshiaki Iba et al. [15] shows that patients with severe forms of the disease have significantly increased fibrinogen levels and a doubling of von Willebrand factor concentrations compared to normal limits. These changes reflect an intense activation of hemostasis and direct damage to the vascular endothelium. The combination of systemic inflammation, endothelial dysfunction and imbalance between pro- and anticoagulant mechanisms favors the occurrence of thrombotic events, reported in hospitalized patients with COVID-19. In this context, early recognition of the thrombotic profile becomes essential, and the data obtained support the early and carefully monitored use of anticoagulant therapy as a strategy to reduce mortality [16].

In a global cohort study of 767 patients co-infected with tuberculosis (active or history) and COVID-19, the cumulative mortality was 11%, with significantly higher rates in Europe (14.2% vs. 9.2%), and multivariable analysis identified older age, male gender, and the need for invasive mechanical ventilation as significant independent risk factors for death [17]. At the same time, complement activation, neutrophil degranulation and platelet activation contribute to the formation of disseminated microthrombi [18]. Additional factors, such as obesity and prolonged immobilization, complete Virchow’s triad, increasing the susceptibility to thrombosis [19]. This state of hypercoagulability is responsible for the increased incidence of arterial and venous thrombotic events, which can lead to fatal cardiovascular complications [20].

Despite the application of standard anticoagulant prophylaxis, the incidence of venous thromboembolic events remains significant in hospitalized patients with moderate and severe forms, especially in intensive care units [21]. These patients have higher mortality rates, requiring an individualized therapeutic approach. In contrast, in patients with mild forms, the thrombotic risk seems much reduced, especially in those fully vaccinated against SARS-CoV-2.

## 3. Problem Statement and Objectives of the Paper

Our hypothesis is that patients with microbiologically confirmed pulmonary tuberculosis who develop SARS-CoV-2 infection during hospitalization may have a higher in-hospital thrombotic risk profile and a higher incidence of thrombotic events compared with patients hospitalized for tuberculosis alone. Understanding the mechanisms of coagulopathy generated by this association could contribute to better clinical management of these patients, through adapted anticoagulation strategies and through more rigorous surveillance of thromboembolic complications. At the same time, clarifying these aspects would have a major impact on public health policies, especially in regions with a high burden of tuberculosis, where medical resources are limited. However, the current literature is extremely poor: there are no controlled studies evaluating patients hospitalized with both diseases concurrently, and information about the thrombotic risk associated with this comorbidity is fragmentary and insufficient. This lack of knowledge is even more problematic as tuberculosis remains endemic in many countries with a low level of development, precisely where the COVID-19 pandemic has exerted additional pressure on health systems.

The primary objective of this study was to explore differences in thrombotic risk and the occurrence of in-hospital thrombotic events between patients with microbiologically confirmed pulmonary tuberculosis, who developed SARS-CoV-2 infection during hospitalization and patients hospitalized with tuberculosis alone. Secondary objectives were to compare clinical course indicators (e.g., length of hospitalization, oxygen requirement), laboratory markers related to coagulation and inflammation, and to examine the relationship between validated thrombotic risk scores (Padua and IMPROVE-DD) and in-hospital thrombotic events. In-hospital mortality was evaluated as an exploratory outcome given the limited sample size and event rate. A COVID-19-only comparison group was not included, which is acknowledged as a limitation.

## 4. Materials and Methods

We conducted a retrospective study, conducted on patients hospitalized in the Adult TB Department of the Pneumology Clinic of the Mures County Clinical Hospital, with the approval of the institution’s Ethics Committee, no. 13903/9 October 2025. The study included two groups of patients: the first group includes adult patients diagnosed with microbiologically confirmed pulmonary tuberculosis and who were infected with the SARS-CoV-2 virus during hospitalization; and the second group of patients with microbiologically confirmed tuberculosis, without a history of COVID-19. The TB-only control group consisted of eligible TB admissions during a comparable calendar period, selected to minimize seasonal and institutional bias. The analyzed period covered the period 2021–2023. The inclusion criteria were microbiological confirmation of tuberculosis, confirmation by RT-PCR or antigenic test of infection with the SARS-CoV-2 virus, in those with co-infection. Patients with incomplete medical records and those without all laboratory tests included in the study were excluded from the study.

For each patient included in the study, clinical, paraclinical and anamnestic data were collected from observation sheets and electronic medical records. The information included demographic data (name, sex, age, environment of origin), details regarding hospitalization (date of admission, date of discharge, duration of hospitalization) and characteristics of the tuberculosis case (bacteriological detection, type of lesion: cavitary or nodular, new case or reactivation). Anthropometric data were also recorded: height, weight, body mass index and behavioral risk factors, such as smoking, alcohol consumption and personal pathological history. Comorbidities were noted separately, including chronic lung diseases, cardiovascular diseases, active cancer, HIV infection and other associated pathologies.

In-hospital thrombotic events included deep vein thrombosis (DVT) and pulmonary embolism (PE). DVT was diagnosed based on clinical suspicion and confirmed by Doppler ultrasonography. PE was clinically suspected and, when available, confirmed by computed tomography pulmonary angiography (CTPA). In this cohort, confirmatory CTPA was available for one PE case. Thrombotic history (“history of thrombosis”) referred to thrombotic events documented prior to the index hospitalization.

Biological parameters analyzed included platelet count, serum fibrinogen levels, D-dimers, transaminases (GOT/AST, GPT/ALT). For patients with SARS-CoV-2 infection, the results of RT-PCR or antigen tests, the severity of the disease (mild, moderate, severe), the need for oxygen therapy, the treatments administered (antivirals, corticosteroids, anticoagulants), as well as the type and duration of associated antibiotic therapy were recorded. Anticoagulation was recorded as treatment present prior to thrombotic events, without consistent documentation allowing differentiation between prophylactic and therapeutic dosing or precise timing.

To classify the severity of SARS-CoV-2 infection, patients were classified into three clinical categories: mild, moderate and severe, depending on the clinical picture, biological parameters and imaging changes. Mild forms included patients with mild respiratory symptoms, no signs of respiratory failure and oxygen saturation values SpO_2_ ≥ 94% in ambient air, in the absence of significant radiological pulmonary infiltrates. Moderate forms were defined by the presence of fever, persistent cough and moderate dyspnea, associated with limited radiological pulmonary infiltrates (below 50% of the parenchyma) and saturation between 90 and 93%. Severe forms were characterized by marked dyspnea, hypoxemia SpO_2_ ≤ 90%, tachypnea >30 breaths/minute and extensive radiological images of bilateral pneumonia, requiring oxygen therapy and, in some cases, ventilatory support. The classification was performed according to clinical and paraclinical criteria recommended by the World Health Organization guidelines. Antituberculosis treatment was detailed by the doses administered of isoniazid (HIN), rifampicin (RMP), pyrazinamide (PZM) and ethambutol (EMB). Surgical history and chronic treatments for pre-existing pathologies were also recorded. The patients’ status at discharge was also assessed, with a focus on mortality rate.

In the database analysis process, we used 3 scores to calculate thrombotic risk, scores that were calculated retrospectively during data analysis to correlate them with thrombotic risk and the need for anticoagulant treatment.

To assess thromboembolic risk in hospitalized patients with tuberculosis, with or without concomitant COVID-19 infection, the Padua score, a validated tool for stratifying the risk of venous thromboembolism in hospitalized patients, was used. The score includes the following criteria: active cancer (3 points), history of venous thrombosis (3 points), prolonged immobilization (3 points), cardiac or respiratory failure (1 point), age ≥ 70 years (1 point) and obesity (BMI ≥ 30 kg/m^2^, 1 point). For each patient, the values were extracted from the observation sheet and summed according to the original guideline. A score ≥ 4 was considered high risk of thrombosis, indicating the need for anticoagulant prophylaxis, and a score < 4 meaning low risk.

Thrombotic risk was also estimated by the IMPROVE-DD score, an extension of the IMPROVE score that integrates the D-dimer value. The criteria included were history of VTE (3 points), active cancer (2 points), immobilization ≥ 7 days (1 point) and age ≥ 60 years (1 point). For the D-dimer component, the ratio between the determined value and the upper limit of normal (ULN) was calculated which was considered 500 ng/mL (FEU), expressed as D-Dimer xULN = value/ULN. Patients with an IMPROVE score ≥ 4 or a score of 2–3 associated with D-dimer > 2 × ULN were classified as having an increased risk of thromboembolic events. This method allows a combined assessment of clinical and biological factors, increasing the accuracy of risk prediction.

For an additional and simplified assessment of thrombotic risk, adapted to the specific context of tuberculosis patients, a rapid mini score was calculated, based on the parameters available in the database. The score included: active cancer (2 points), reduced mobility (2 points), age ≥ 70 years (1 point), BMI ≥ 30 kg/m^2^ (1 point), D-dimer > 2 × ULN (2 points) and severe form of COVID-19 (1 point). The rapid mini score was evaluated as an exploratory, non-validated tool, derived from variables available in the dataset and was not used to support primary conclusions. Its results were interpreted cautiously and primarily as hypothesis-generating.

All statistical analyses were performed using GraphPad Prism, version 10.4.1 (GraphPad Software, Boston, MA, USA). Continuous variables were assessed for normality using the Shapiro–Wilk test. Normally distributed variables were reported as mean ± standard deviation and compared using the independent samples *t*-test, while non-normally distributed variables were expressed as median and interquartile range and compared using the Mann–Whitney U test.

Categorical variables were presented as absolute numbers and percentages, and differences between groups were evaluated using the chi-square test or Fisher’s exact test when expected cell counts were <5. Unadjusted odds ratios (ORs) with corresponding 95% confidence intervals (CIs) were calculated to estimate associations between categorical variables and outcomes.

The primary outcome was the occurrence of in-hospital thrombotic events, defined as clinically suspected deep vein thrombosis supported by Doppler ultrasonography, with confirmatory computed tomography pulmonary angiography available for cases of suspected pulmonary thromboembolism.

Associations between risk score categories (Padua, IMPROVE-DD, and the rapid mini-score) and the occurrence of in-hospital thrombotic events were examined using 2 × 2 contingency tables, with odds ratios (ORs) and 95% confidence intervals (CIs) calculated accordingly. Fisher’s exact test was used to determine statistical significance for these comparisons. A two-tailed *p* value below 0.05 was considered statistically significant.

Given the limited number of thrombotic events, multivariable analysis was performed using Firth penalized logistic regression to reduce small sample bias. A minimally adjusted model including age and TB-COVID-19 coinfection status was selected a priori to limit overfitting.

## 5. Results

### 5.1. Demographic and Lifestyle Characteristics

Baseline demographic and lifestyle characteristics are presented in Table 1. The two groups had comparable sex distribution, while age differed significantly, with patients in the TB-only group being older (*p* = 0.003). Smoking and alcohol consumption showed no statistically significant differences between groups (all *p* > 0.05).

### 5.2. Tuberculosis-Related Characteristics

Tuberculosis-related characteristics are summarized in Table 1. No significant differences were observed between groups regarding the type of pulmonary involvement (cavitary vs. non-cavitary) or the proportion of newly diagnosed TB cases versus reactivation (all *p* > 0.05).

### 5.3. Comorbidities

As shown in Table 1, most comorbidities, including cardiovascular disease, malignancies, HIV infection, and prior thrombotic events, occurred at similar frequencies (all *p* > 0.05). Chronic respiratory disorders were the only comorbidity significantly more frequent in the TB-COVID-19 coinfection group compared with the TB-only group (80% vs. 52.5%, *p* = 0.01).

### 5.4. Clinical Status at Admission

Clinical status at admission is detailed in Table 1. Respiratory insufficiency, reduced mobility, and COVID-19 vaccination status did not differ significantly between groups (all *p* > 0.05).

### 5.5. Risk Stratification Scores

Thrombotic risk stratification scores are presented in Table 1. A high Padua score was significantly more frequent in the TB-COVID-19 coinfected group (55% vs. 20%, *p* = 0.002). The IMPROVE-DD score also showed a higher proportion of high-risk classifications in this group, with values close to the conventional statistical significance threshold (*p* = 0.07). In contrast, the rapid mini score did not differ significantly between groups (*p* = 0.40).

### 5.6. Association Between Padua and IMPROVE-DD Scores and In-Hospital Thrombotic Events

Within-group analyses examining the association between thrombotic risk scores and in-hospital thrombotic events are described below; detailed cross-tabulations are available upon request. In the TB-COVID-19 coinfected group, a high Padua score was significantly associated with the development of in-hospital thrombotic events (OR = 9.71, 95% CI: 1.08–87.32; *p* = 0.02). A similar association was observed in the TB-only group, where patients classified as high risk also demonstrated significantly higher odds of thrombosis during hospitalization (OR = 18.6, 95% CI: 1.60–216.07; *p* = 0.02).

For the IMPROVE-DD score, the analysis conducted within the TB-COVID-19 subgroup approached statistical significance, with higher odds of thrombotic events among patients categorized as high risk (OR = 5.75, 95% CI: 0.95–34.79; *p* = 0.08).

### 5.7. Hospitalization Characteristics

Length of hospitalization, summarized in Table 2, was significantly longer in the TB-only group (*p* = 0.012). Antibiotic therapy (Table 1) was more frequently administered in the TB-COVID-19 group, with values close to the conventional threshold for statistical significance (*p* = 0.06). Oxygen supplementation (Table 1) was numerically more frequent in the TB-COVID-19 group; however, this difference did not reach statistical significance (*p* = 0.18). The use of anticoagulation prior to thrombotic events did not differ significantly between groups (*p* = 0.35). In-hospital thrombotic events and mortality occurred at similar rates in both groups (all *p* > 0.05).

### 5.8. Laboratory Findings

Laboratory parameters are presented in Table 2. Serum transaminases (AST and ALT) and fibrinogen did not differ significantly between groups. D-dimer values approached but did not reach statistical significance (*p* = 0.07). Platelet counts differed significantly, with higher values in the TB-only group (*p* = 0.001).

### 5.9. Multivariable Analysis of In-Hospital Thrombotic Events

Results of the multivariable analysis are presented in Table 3. After adjustment for age using Firth penalized logistic regression, TB-COVID-19 coinfection remained associated with higher odds of in-hospital thrombotic events; however, this association did not reach statistical significance and was characterized by wide confidence intervals, reflecting limited statistical power.

## 6. Discussion

### 6.1. Demographic and Lifestyle Characteristics

Sex distribution was comparable between groups, without statistically significant differences. However, tuberculosis patients without COVID-19 were significantly older than those coinfected. This aspect could influence the subsequent interpretation of thrombotic risk, since age is an independent risk factor for venous thromboembolism [15].

Body mass index was similar between the two groups, with both groups falling into the underweight range. This is a precarious nutritional profile typical of tuberculosis patients. It reflects both the immunological vulnerability and the increased susceptibility to acute respiratory infections of these patients. However, the absence of statistically significant differences between groups suggests that nutritional status does not explain the observed variations in thrombotic risk. SARS-CoV-2 vaccination status was similar between groups. The small number of vaccinated patients limits the study’s ability to evaluate the effects of vaccination on the severity of clinical forms of COVID-19 and limits the ability to evaluate the potential effect of vaccination on thrombotic risk.

Tobacco and alcohol consumption did not differ significantly between groups, suggesting that these behaviors do not explain the differences in thrombotic risk observed. However, in both groups the percentage of smokers is high and the cumulative consumption expressed in pack-years does not differ significantly between groups. This confirms smoking as a common risk factor for both groups, without a discriminatory role on thrombotic risk. Given that smoking is recognized as one of the main risk factors for tuberculosis, this fact may explain the increased incidence of this disease in Romania [22], but does not explain the differences in the procoagulant profile observed in the context of coinfection with the SARS-CoV-2 virus. The percentage of chronic alcohol consumers is quite high in both groups, but still much lower than the percentage of smokers. Alcoholism leads to immunodepression due to the liver damage it triggers [23], which may explain both the susceptibility of patients in the group to infection with Koch’s bacillus and to infection with the SARS-CoV-2 virus. The prevalence of both alcohol consumption and smoking, in the group, is visually represented in Supplementary Figure S1.

### 6.2. Duration of Hospitalization

The duration of hospitalization differed significantly between the two groups of patients, with those with tuberculosis and without COVID-19 having a longer hospitalization period compared to those with co-infection. This difference can be explained by the evolutionary nature of tuberculosis, which typically requires a longer period of monitoring and clinical stabilization, especially in patients with infiltrative or cavitary forms, as well as by the need to evaluate the response to anti-tuberculosis therapy. In contrast, in the co-infected group, clinical management was influenced by the acute nature of the SARS-CoV-2 infection, which often requires intensive surveillance in a shorter interval, but not necessarily an extension of the total duration of hospitalization.

### 6.3. Tuberculosis Characteristics

Both the type of lung involvement, in our study cavitary and nodular involvement, and the proportion of new cases compared with tuberculosis reactivations, were similar between groups. Thus, the anatomopathological severity of tuberculosis did not differ significantly, suggesting that the thrombotic evolution is influenced by the overlap of infection with the SARS-CoV-2 virus, not by the form or stage of pulmonary tuberculosis.

### 6.4. Comorbidities

The comorbidities considered in both groups (see Supplementary Figure S2), known as major risk factors for thrombotic events: cardiovascular disease, active neoplasia, HIV infection and history of thrombotic events occurred with similar frequencies in the two groups. This distribution of the data indicates that the differences in thrombotic risk are not explained by the profile of pre-existing comorbidities, but rather by the additional impact exerted by SARS-CoV-2 infection on the coagulation status. The most statistically relevant comorbidities were chronic lung diseases, which were significantly more frequent in the co-infected group. Chronic lung diseases have been associated with severe forms of COVID-19 and also represent a risk factor for any respiratory intercurrent by decreasing the defense capacity of the respiratory epithelium, due to chronic inflammation [24]. The difference between the two groups compared reveals the increased respiratory vulnerability of co-infected patients, possibly explaining the increased need for oxygen therapy and the high thrombotic risk.

### 6.5. Clinical Status and Therapeutic Need

Respiratory failure and reduced mobility did not differ statistically between groups, reflecting a comparable level of functional impairment. Oxygen therapy was numerically more frequent in the TB-COVID-19 coinfected group, although the difference did not reach statistical significance. Antibiotic therapy was also more commonly used in coinfected patients, showing a directionally consistent effect, although not statistically significant. These patterns may reflect greater clinical concern for acute deterioration or bacterial superinfection during SARS-CoV-2 infection. However, given the exploratory nature of the study, these observations should be interpreted cautiously.

The use of anticoagulation, either for prophylactic or therapeutic purposes, did not differ significantly between the two groups, despite a higher thrombotic risk suggested by the Padua and IMPROVE-DD scores in the co-infected cohort. This incongruence between the estimated risk profile and the applied therapeutic conduct reflects the retrospective nature of the study and the calculation of these risk scores. At the same time, the lack of a standardized assessment of thromboembolic risk in current medical practice is emphasized, suggesting the need to implement uniform stratification and prophylaxis protocols.

### 6.6. Laboratory Analyses

In terms of biochemical markers, transaminase values were similar between the two groups, with no statistically significant differences between the AST and ALT values of the two groups. Thus, liver damage cannot be considered a distinguishing element between patients with simple tuberculosis and those with tuberculosis and COVID-19 coinfection. The variability of transaminase values can be considered a reflection of the systemic inflammatory syndrome associated with the two types of infections or an adverse effect of antituberculosis medication.

Fibrinogen values were higher in the group with isolated tuberculosis, but without having statistical significance. Due to the small sample size and high variability, it cannot be firmly concluded that there is a real difference between the two groups of patients. Both Koch bacillus infection and SARS-CoV-2 virus infection represent pathologies with an important inflammatory profile, through persistent chronic inflammation, respectively, through acute inflammation and cytokine storm. Therefore, the values between the two groups were similar, and fibrinogen, being an acute phase protein, seems not to effectively discriminate hypercoagulability in the context of coinfection, in contrast to other biomarkers such as D-dimers [21].

Thrombotic risk stratification by validated clinical scores (particularly Padua) was more frequently elevated in the coinfected group. This supports the hypothesis of an additive prothrombotic risk profile in TB-COVID-19 coinfection. This observation is consistent with the pathophysiological mechanisms of coagulopathy associated with SARS-CoV-2 virus infection, characterized by intense activation of the coagulation cascade, endothelial dysfunction and excessive formation of thrombin and microthrombi [25].

As platelet levels were higher in the tuberculosis group, a possible explanation could be the persistent chronic inflammation. This is associated with tuberculosis and known for the clinical picture in which reactive thrombocytosis is often present. In accordance with the pathogenesis of the SARS-CoV-2 virus, which, through peripheral consumption, induces a decrease in platelet count and thrombocytopenia, patients in the tuberculosis-COVID-19 coinfection group reported lower platelet levels. This difference between the two groups highlights the contrast between the chronic inflammation of tuberculosis and the acute inflammation of the viral infection. The differences between laboratory findings in the group are synthetized in Supplementary Figure S3 (see Supplementary Materials).

Overall, laboratory parameters did not demonstrate statistically significant between-group differences, except for platelet counts. D-dimer values approached statistical significance; however, given the limited sample size and variability, no firm conclusions regarding their discriminative value can be drawn. These findings should therefore be interpreted cautiously and within the exploratory framework of the study.

### 6.7. Major Clinical Events: Thrombosis and Mortality

Importantly, SARS-CoV-2 infection occurred during hospitalization and represents a time-dependent exposure. As the analysis did not use time-to-event modeling, comparisons may be affected by immortal time and selection bias and results should therefore be interpreted with caution.

The estimated odds ratio suggests a numerically higher risk of thrombotic events in the group with tuberculosis and COVID-19 co-infection. These findings are consistent with the literature, where co-infection between tuberculosis and SARS-CoV-2 is associated with significant systemic inflammation, endothelial dysfunction and activation of the coagulation cascade, which together contribute to the occurrence of thrombotic risk [26]. Similarly, the overlap of SARS-CoV-2 infection over a pre-existing inflammatory context, such as active tuberculosis, is emphasized, which amplifies hypercoagulability and predisposes patients to thrombotic phenomena, a fact supported by the literature [4].

Importantly, patients in the TB-only group were significantly older, which could confound comparisons of thrombotic risk. To address this, we performed an age-adjusted multivariable analysis using Firth penalized logistic regression given the limited number of thrombotic events. TB-COVID-19 coinfection remained associated with higher odds of in-hospital thrombotic events, although estimates were imprecise with wide confidence intervals, underscoring the exploratory nature of these findings.

In-hospital mortality did not differ significantly between groups. Given the limited sample size and low number of deaths, mortality analyses are exploratory and the study is not powered to detect modest between-group differences. Nevertheless, the numerically higher mortality in the coinfected group is consistent with published cohorts [27] reporting worse clinical trajectories in TB-COVID-19 coinfection, warranting confirmation in larger prospective studies. These support that patients with tuberculosis and COVID-19 coinfection tend to have a more severe course and to require oxygen therapy more frequently, due to extensive lung involvement and due to the higher risk of rapid decompensation [28]. For visual clarity, see Supplementary Figure S4, which compares the proportion of patients with thrombotic events and mortality in the two groups, illustrating the observed differences and supporting the statistical and clinical interpretation of these results.

### 6.8. Interpretation of Thrombotic Risk Scores (PADUA, IMPROVE-DD, Mini-Score)

For a retrospective assessment of thrombotic risk in the patient cohort, we used three scores: Padua, Improve-DD and calculated a rapid mini-score. Statistical analysis of these scores showed significant differences between the two groups of patients. Of the three scores used, the Padua score (see Supplementary Figure S5) proved to be the most statistically significant, this being a score already validated in the specialty literature as a tool used in the stratification of thrombotic risk of hospitalized patients [29]. These stable and reproducible differences, despite the small sample size, suggest a possible additive prothrombotic effect of SARS-CoV-2 infection. In the co-infection group, a high Padua score was associated with the occurrence of thrombotic phenomena during hospitalization. In the tuberculosis group, a similar association was observed with an increased risk of thrombotic phenomena.

The IMPROVE-DD score showed an increased risk of thrombotic events of 37.5% in the co-infection group versus 17.5% in the tuberculosis group. The score showed a higher proportion of high-risk classification in the coinfected group, driven primarily by clinical components of the score rather than absolute D-dimer levels. Although the numerical difference between the two groups is important, statistical significance was not reached. Although the *p*-value did not reach the conventional threshold for statistical significance, the direction of effect was consistent with that observed for the Padua score. Given the limited sample size, these findings should be interpreted cautiously and considered exploratory. The results of the mini-rapid score did not reveal significant differences between the two groups, which can also be explained by a lower sensitivity of this score compared to the other validated scores. The distribution of scores was relatively similar in the two groups, and statistical analysis indicates that the instrument does not have the power to sufficiently discriminate thrombotic risk in the conditions of this study (see Supplementary Figure S6). This result may reflect both the limitations of the score and the structure of the dataset, in which the variables included in the mini score had homogeneous distributions and similar prevalences between groups.

It is important to mention that the risk score calculations were performed retrospectively, within the post hoc analysis, exclusively for research purposes, without influencing medical decisions or therapeutic conduct of the patients included in this study. Although an age-adjusted logistic regression using Firth penalized methods was performed, the limited number of thrombotic events restricted the number of covariates that could be included. Therefore, associations between risk scores and thrombotic events may still be influenced by residual confounding. However, risk scores (Padua and IMPROVE-DD) were more frequently elevated in the co-infected group, suggesting that the presence of SARS-CoV-2 infection exerts an additional, age-independent effect on hypercoagulability.

## 7. Clinical Implications and Future Research

The results of the study emphasize the importance of early assessment of thrombotic risk in patients coinfected with tuberculosis and SARS-CoV-2, given the numerically higher incidence of thromboembolic events in this category. Systematic assessment of thrombotic risk may be considered in patients with TB-COVID-19 coinfection, particularly in high-risk clinical contexts; however, prospective validation is required before definitive clinical recommendations can be made. It is also necessary to consider an adapted anticoagulant prophylaxis, according to validated risk scores (Padua, IMPROVE-DD), especially in patients with severe forms of the disease or multiple comorbidities. In the context of countries with high prevalence of tuberculosis and high COVID-19 caseload, the implementation of standardized protocols for the prevention and monitoring of thrombotic complications could contribute to reducing mortality and improving the prognosis of these vulnerable patients.

To better understand the interaction between tuberculosis, SARS-CoV-2 infection and thrombotic risk, prospective multicenter studies are needed, including larger cohorts and populations from different geographical regions. Future investigations should aim to elucidate the pathophysiological mechanisms involved, by analyzing coagulation and inflammation biomarkers to identify key factors contributing to coagulopathy associated with coinfection. Controlled clinical trials are also needed to evaluate the efficacy and safety of prophylactic and therapeutic anticoagulation strategies in coinfected patients, with the aim of defining personalized treatment protocols. Integration of these data could allow the development of predictive models and dedicated clinical guidelines, aimed at reducing morbidity and mortality associated with this complex pathological association.

## 8. Limitations of the Study

This study has limitations inherent to its retrospective, single-center design, which restricts generalizability and precludes causal inference. The relatively small sample size and low number of thrombotic events limited statistical power and increased the risk of type II error, resulting in wide confidence intervals around effect estimates. Therefore, all findings should be interpreted as exploratory and hypothesis-generating.

Diagnosis of thrombotic events relied primarily on clinical suspicion supported by Doppler ultrasonography, while confirmatory CTPA for pulmonary embolism was available only in a single case. Limited access to systematic imaging may have led to underdiagnosis (particularly of asymptomatic DVT) and outcome misclassification. Consequently, the true incidence of thrombotic events may be underestimated, and between-group comparisons may be affected by diagnostic variability.

SARS-CoV-2 infection occurred during hospitalization and therefore represents a time-dependent exposure. Because the analysis treated coinfection status as a fixed exposure and did not use time-to-event modeling, immortal time and selection bias cannot be excluded. Also, laboratory tests were not uniformly available for all patients, and the impossibility of genotyping the viral subtypes of SARS-CoV-2 prevented the assessment of possible differences between viral variants in terms of the severity of coagulopathy. Another important limitation is related to the fact that the COVID-19 pandemic has ended, and currently testing for SARS-CoV-2 infection is no longer routinely performed, which significantly reduces the possibility of extending the study to a larger group and affects the comparability of recent data.

Although an age-adjusted multivariable analysis using Firth penalized logistic regression was performed, the limited number of thrombotic events restricted the number of covariates that could be included without overfitting. Residual confounding by comorbidities and clinical severity may therefore persist. In this context, variantion in coagulation-related markers, together with the high prevalence of Padua scores ≥ 4 and IMPROVE-DD ≥ 4, raise the possibility that some thrombotic events may not have been clinically detected. In particular, asymptomatic deep vein thromboses may have remained undiagnosed in the absence of systematic imaging.

At the same time, Romania remains an endemic country for tuberculosis, which favors a high incidence, but also an underdiagnosis of cases with little symptoms or limited access to medical services, an aspect that may introduce a selection bias in the analyzed cohort. In the absence of a post-discharge follow-up, it was not possible to assess long-term thrombotic risk. Despite all these limitations, the study provides a relevant and necessary perspective on the interaction between tuberculosis, COVID-19 infection and thrombotic risk, in a particular epidemiological context for Romania.

## 9. Conclusions

In our retrospective single-center study cohort, TB-COVID-19 coinfection was associated with a higher thrombotic risk profile, reflected by a significantly higher proportion of high Padua scores and with numerically higher rates of in-hospital thrombotic events compared with TB alone, without reaching statistical significance. Age-adjusted analysis suggested higher odds of thrombotic events in coinfected patients, but estimates were imprecise due to the limited number of events. These findings should be interpreted as exploratory and hypothesis-generating. Prospective multicenter studies with systematic imaging and robust multivariable adjustment are needed to better quantify thrombotic risk and inform evidence-based thromboprophylaxis strategies in this population.

These findings are in line with the known synergistic pathophysiological interaction between chronic inflammation in tuberculosis and acute endothelial dysfunction and hypercoagulability induced by SARS-CoV-2 infection. The coexistence of these mechanisms could amplify the prothrombotic environment, even in the absence of overt clinical events. From a clinical perspective, our findings highlight the need for systematic assessment of thrombotic risk and the use of scoring tools in patients with active tuberculosis who develop SARS-CoV-2 infection. In tuberculosis-endemic areas, where the burden of COVID-19 remains significant, these measures may contribute to reducing complications and improving patient outcomes. Future large-scale multicenter studies, ideally with prospective design and multivariate adjustment, are essential to validate these associations, determine causality, and refine evidence-based anticoagulation strategies for this vulnerable population.

## Figures and Tables

**Table 1:** Baseline demographic, clinical, and tuberculosis-related characteristics of the study population.

Parameter	GROUP 1 TB-COVID-19 Coinfection *N* = 40		GROUP 2 TB *N* = 40		*p*-Value	OR	95%CI
	*n*	%	*n*	%			
Sex Male Female	32 8	80 20	30 10	75 25	0.78	1.33	0.48 to 4.06
Form of TB: Cavitary Nodular	28 12	70 30	29 11	72.5 27.5	0.99	0.88	0.30 to 2.39
New case Reactivation	32 8	80 20	31 9	77.5 22.5	0.99	1.16	0.39 to 3.63
Smoking Yes No	34 6	85 15	31 9	77.5 22.5	0.56	1.64	0.50 to 5.44
Alcohol consumption Yes No	15 25	37.5 62.5	14 26	35 65	0.99	1.14	0.46 to 2.69
CV diseases Yes No	15 25	37.5 62.5	18 22	45 55	0.65	0.73	0.31 to 1.85
Chronic respiratory disorders Yes No	32 8	80 20	21 19	52.5 47.5	0.01	3.61	1.39 to 10.12
Malignancies Yes No	11 29	27.5 72.5	10 30	25 75	0.99	1.23	0.43 to 2.90
HIV infection Yes No	7 33	17.5 82.5	5 35	12.5 87.5	0.75	1.48	0.44 to 4.65
Thrombosis history Yes No	12 28	30 70	8 32	20 80	0.43	1.71	0.58 to 5.03
Thrombotic events during admission Yes No	9 31	22.5 77.5	4 36	10 90	0.22	2.61	0.73 to 9.32
Respiratory failure Yes No	23 17	57.5 42.5	22 18	55 45	0.99	1.04	0.43 to 2.47
Oxygen need Yes No	21 19	52.5 47.5	14 26	35 65	0.18	2.05	0.82 to 5.13
Reduced mobility Yes No	16 24	40 60	15 25	37.5 62.5	0.99	1.11	0.47 to 2.61
COVID-19 vaccinated Yes No	8 32	20 80	12 28	30 70	0.43	0.58	0.19 to 1.71
Antibiotics use Yes No	19 21	47.5 52.5	10 30	25 75	0.06	2.73	0.99 to 7.52
**Anticoagulation** Yes No	17 23	42.5 57.5	12 28	30 70	0.35	1.73	0.69 to 4.34
Death Yes No	6 34	15 85	4 36	10 90	0.73	1.58	0.44 to 5.30
PADUA score High Risk Low Risk	22 18	55 45	8 32	20 80	0.002	4.88	1.88 to 13.78
IMPROVE-DD score High Risk Low Risk	15 25	37.5 62.5	7 33	17.5 82.5	0.07	2.82	1.00 to 7.23
Mini Quick score High Risk Low Risk	10 30	25 75	6 34	15 85	0.40	1.88	0.62 to 6.10

Data are presented as numbers (*n*) and percentages (%) for categorical variables and as mean ± standard deviation or median with interquartile range for continuous variables, as appropriate. Comparisons between groups were performed using Fisher’s exact test for categorical variables and the Mann–Whitney U test for continuous variables.

**Table 2:** Comparison of continuous demographic, clinical, and laboratory variables between patients with TB-COVID-19 coinfection and TB-only.

Variable	GROUP 1 TB-COVID-19 Coinfection *N* = 40 Mean ± SD/Median [IQR]	GROUP 2 TB-Only *N* = 40 Mean ± SD/Median, IQR	t/U	*p*-Value
Age	54.13 ± 13.55	62.65 ± 11.48	3.03	0.003
Hospitalization days	32.5 [10.75–47.25]	47.5 [31–57.5]	538	0.012
BMI	18.21 [16.99–19.90]	17.48 [15.96–21.66]	739	0.56
PA	22.5 [9–31.75]	24 [10.75–31.5]	792	0.94
GOT/AST	20 [14–40.75]	19.50 [13.50–30.50]	792.5	0.94
GPT/ALT	23.5 [15.5–36.75]	24.50 [18.25–32.50]	694.5	0.31
Fibrinogen (mg/dL)	409 [306–502.5]	450 [359–604]	659	0.17
D-Dimers (ng/mL FEU)	415 [137.3–708]	486.5 [475.3–498.8]	615.5	0.07
Platelets (×10^3^/mm^3^)	330.9 ± 179.7	519.5 [349.5–694.8]	376	0.001

Data are presented as mean ± standard deviation or median (IQR). Between-group comparisons were performed using the Mann–Whitney U test.

**Table 3:** Multivariable analysis of factors associated with in-hospital thrombotic events.

Variable	Adjusted OR	95% CI	*p*-Value
TB-COVID-19 coinfection	3.38	0.91–12.56	0.07
Age (per year increase)	1.03	0.98–1.09	0.21

## Data Availability

The datasets generated and analyzed during the current study are not publicly available due to ethical and privacy constraints but are available from the corresponding author on reasonable request.

## Supplementary Materials

The following supporting information can be downloaded at: https://www.mdpi.com/article/10.3390/diagnostics16050724/s1, Figure S1: Prevalence of chronic smoking and alcohol consumption; Figure S2: Prevalence of comorbidities; Figure S3: Boxplots of laboratory parameters; Figure S4: Mortality and thrombotic events comparison; Figure S5: Padua score distribution; Figure S6: Odds ratios of thrombotic risk scores.

## Abbreviations

The following abbreviations are used in this manuscript:

TB	Tuberculosis
COVID-19	Coronavirus Disease 2019
SARS-CoV-2	Severe Acute Respiratory Syndrome Coronavirus 2
VTE	Venous Thromboembolism
DVT	Deep Vein Thrombosis
PE	Pulmonary Embolism
CAC	COVID-19-associated coagulopathy
CRP	C-reactive protein
IL-6	Interleukin-6
ACE2	Angiotensin-Converting Enzyme 2
BMI	Body Mass Index
IQR	Interquartile Range
SD	Standard Deviation
OR	Odds Ratio
CI	Confidence Interval
ULN	Upper Limit of Normal
AST	Aspartate Aminotransferase
ALT	Alanine Aminotransferase
FEU	Fibrinogen Equivalent Units
RT-PCR	Reverse Transcriptase Polymerase Chain Reaction
HIV	Human Immunodeficiency Virus
ICU	Intensive Care Unit
