# Supplementary figures and images for: Assessment of Thrombotic Risk in Patients with Tuberculosis and SARS-CoV-2 Coinfection: A Retrospective Study
Source: Diagnostics (Basel). 2026 Feb 28;16(5):724. doi: 10.3390/diagnostics16050724 (PMC12984595; doi:10.3390/diagnostics16050724)

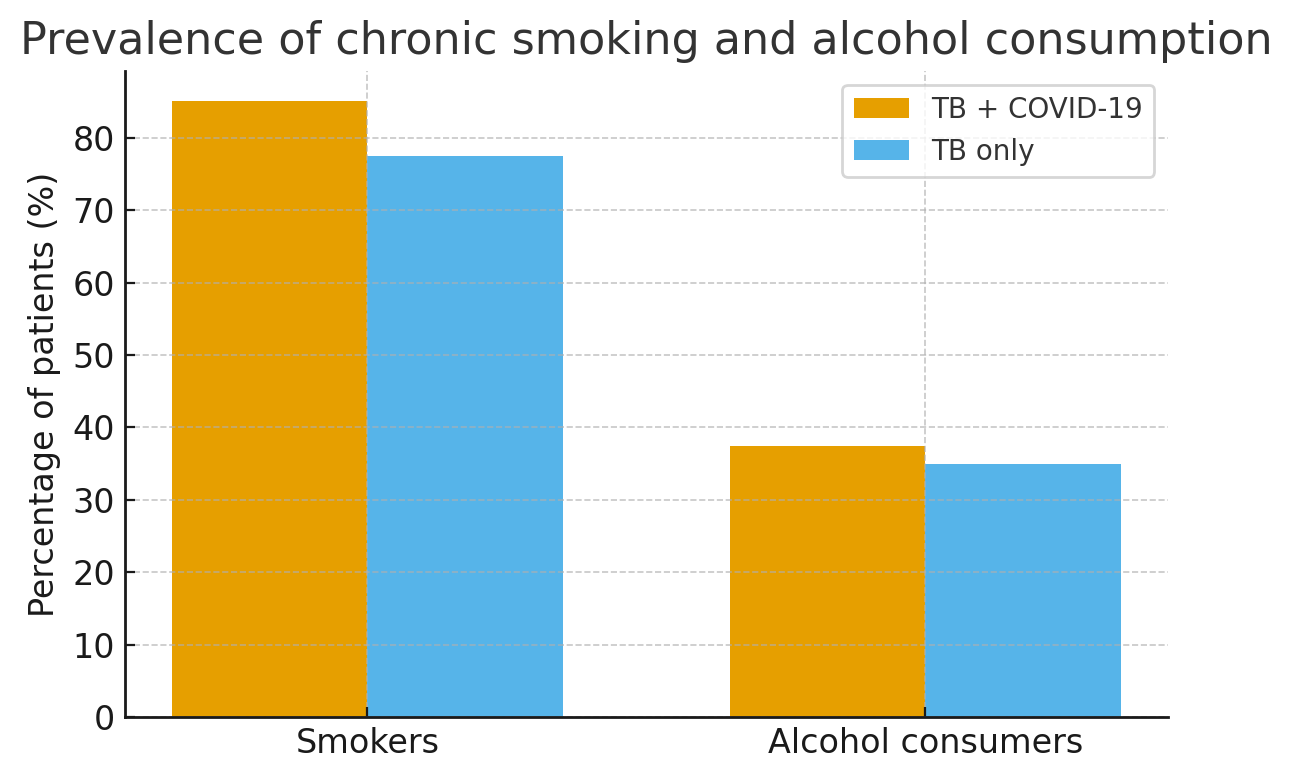

Supplement: Supplementary file 1 [file diagnostics-16-00724-s001.zip › Figure S1.png]

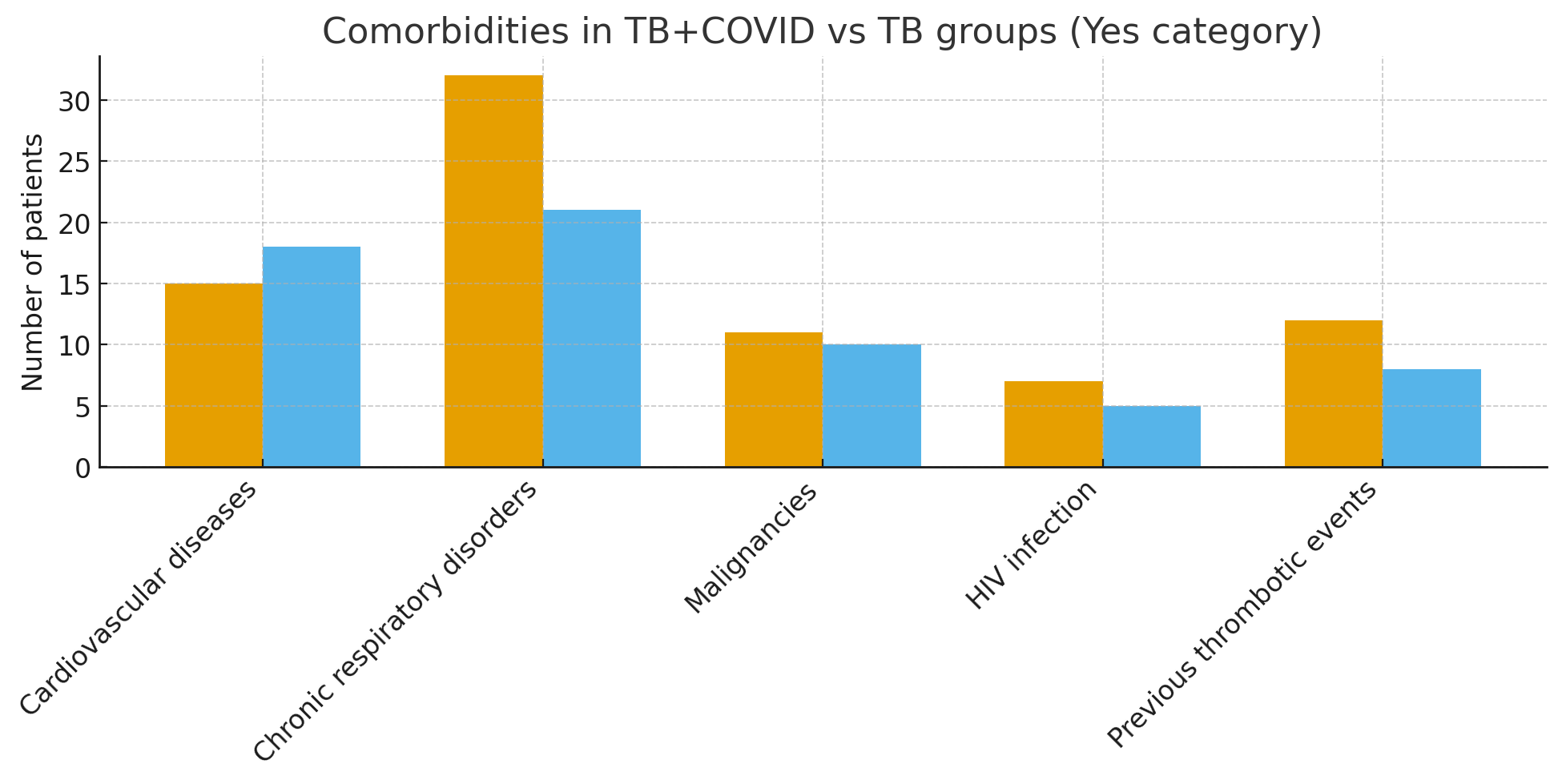

Supplement: Supplementary file 1 [file diagnostics-16-00724-s001.zip › Figure S2.png]

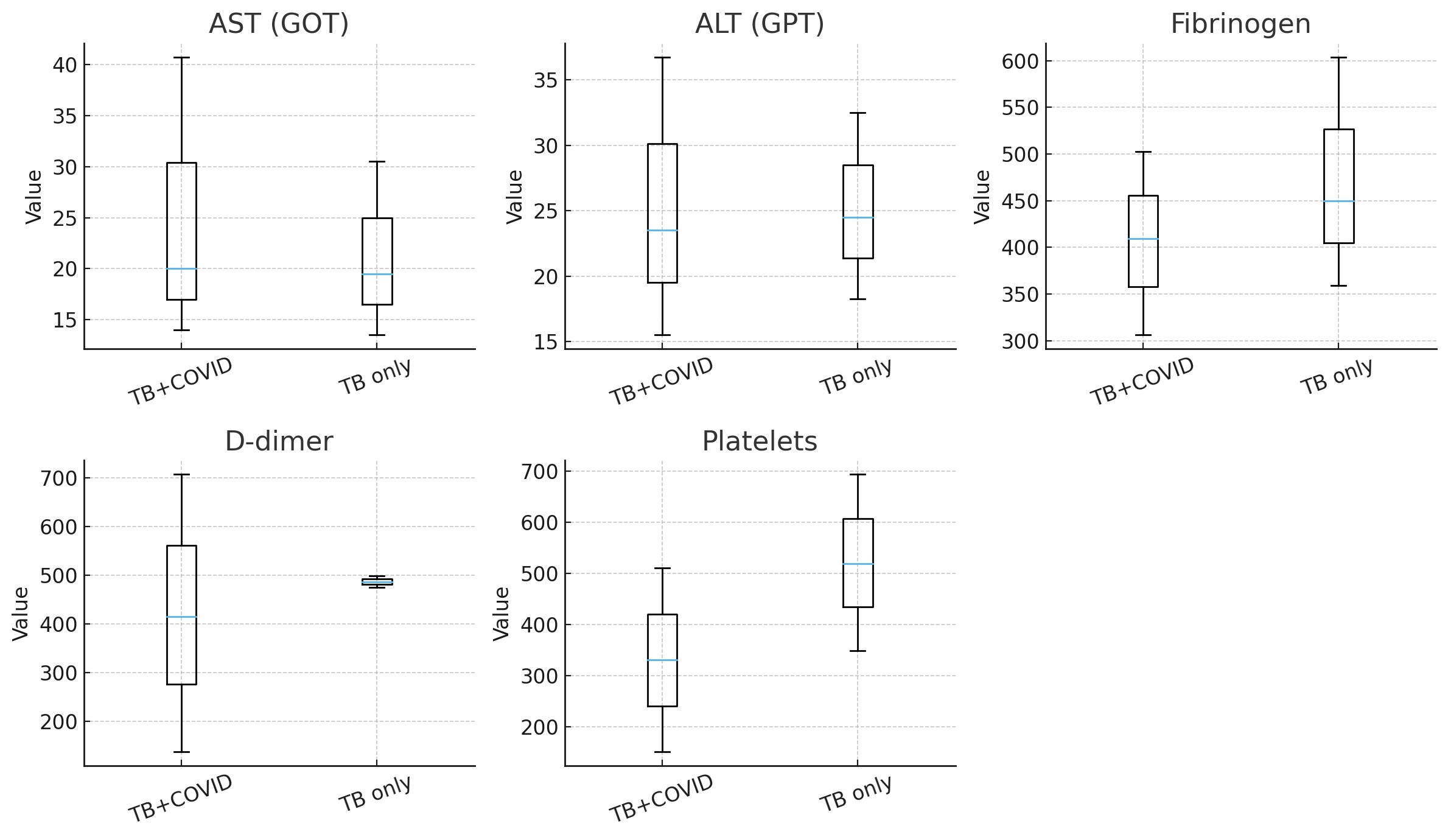

Supplement: Supplementary file 1 [file diagnostics-16-00724-s001.zip › Figure S3.png]

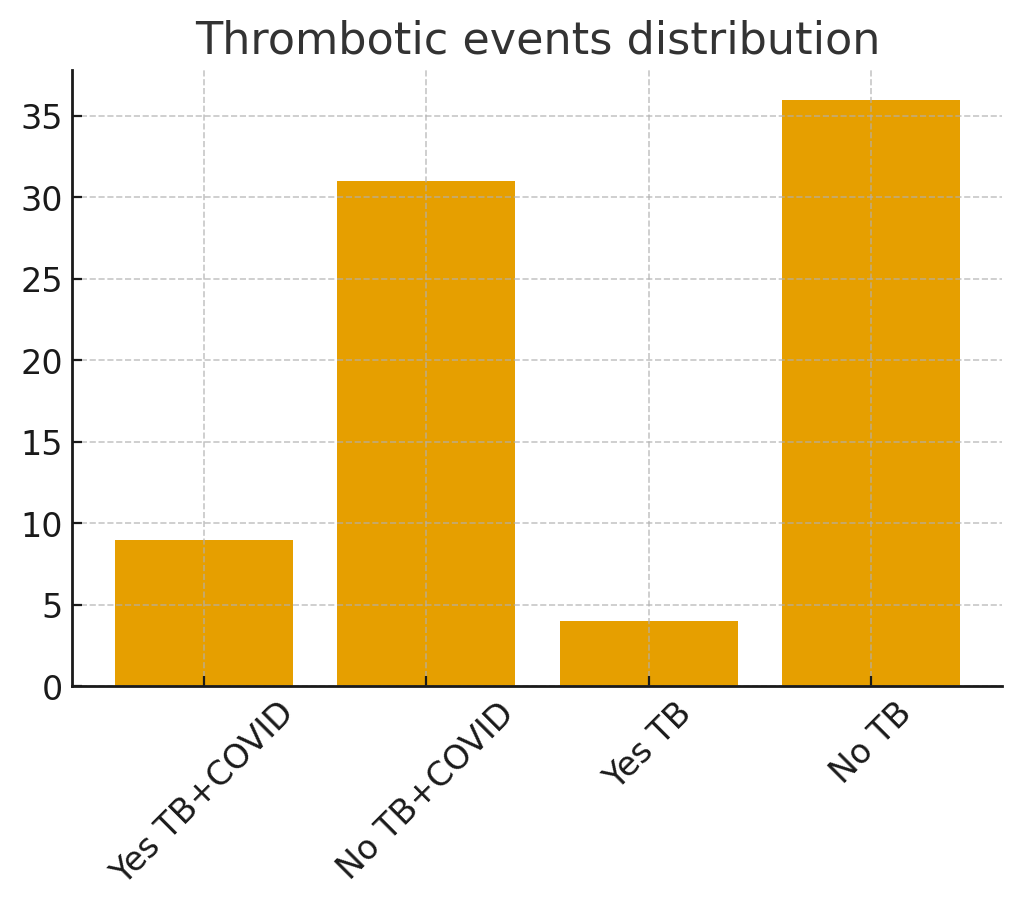

Supplement: Supplementary file 1 [file diagnostics-16-00724-s001.zip › Figure S4.png]

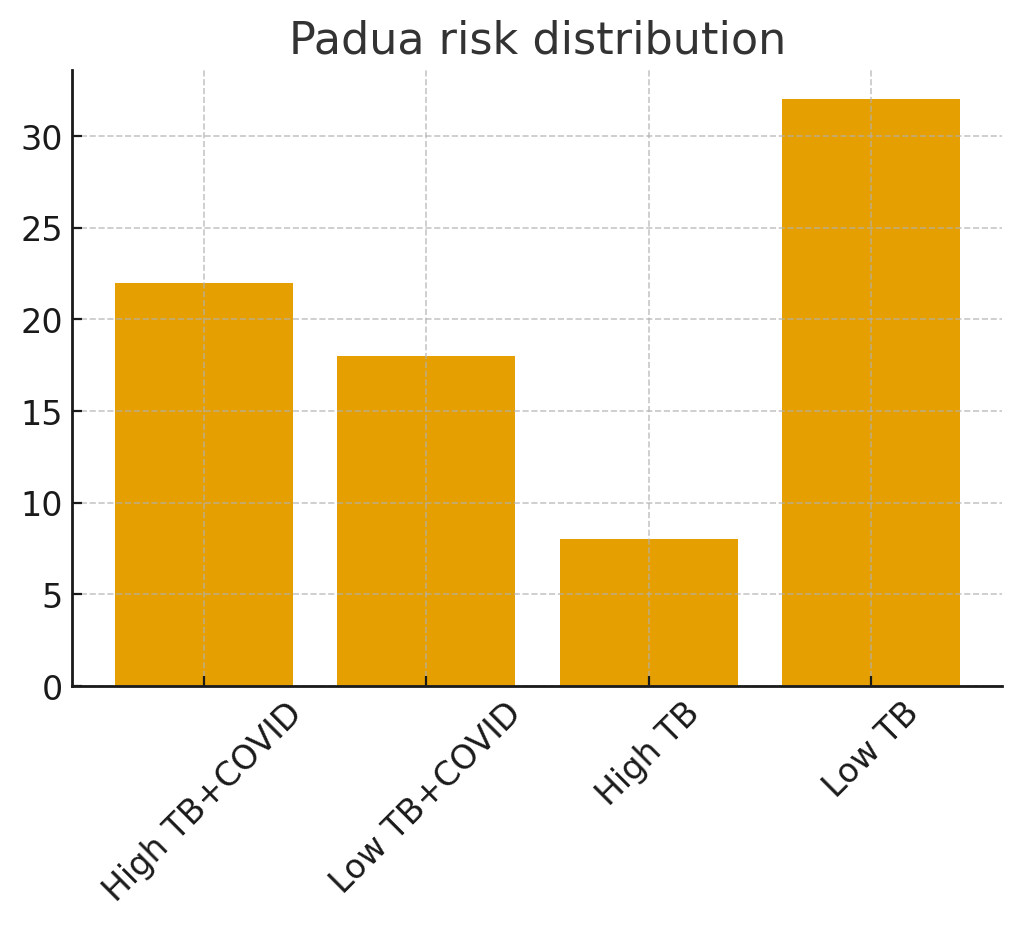

Supplement: Supplementary file 1 [file diagnostics-16-00724-s001.zip › Figure S5.png]

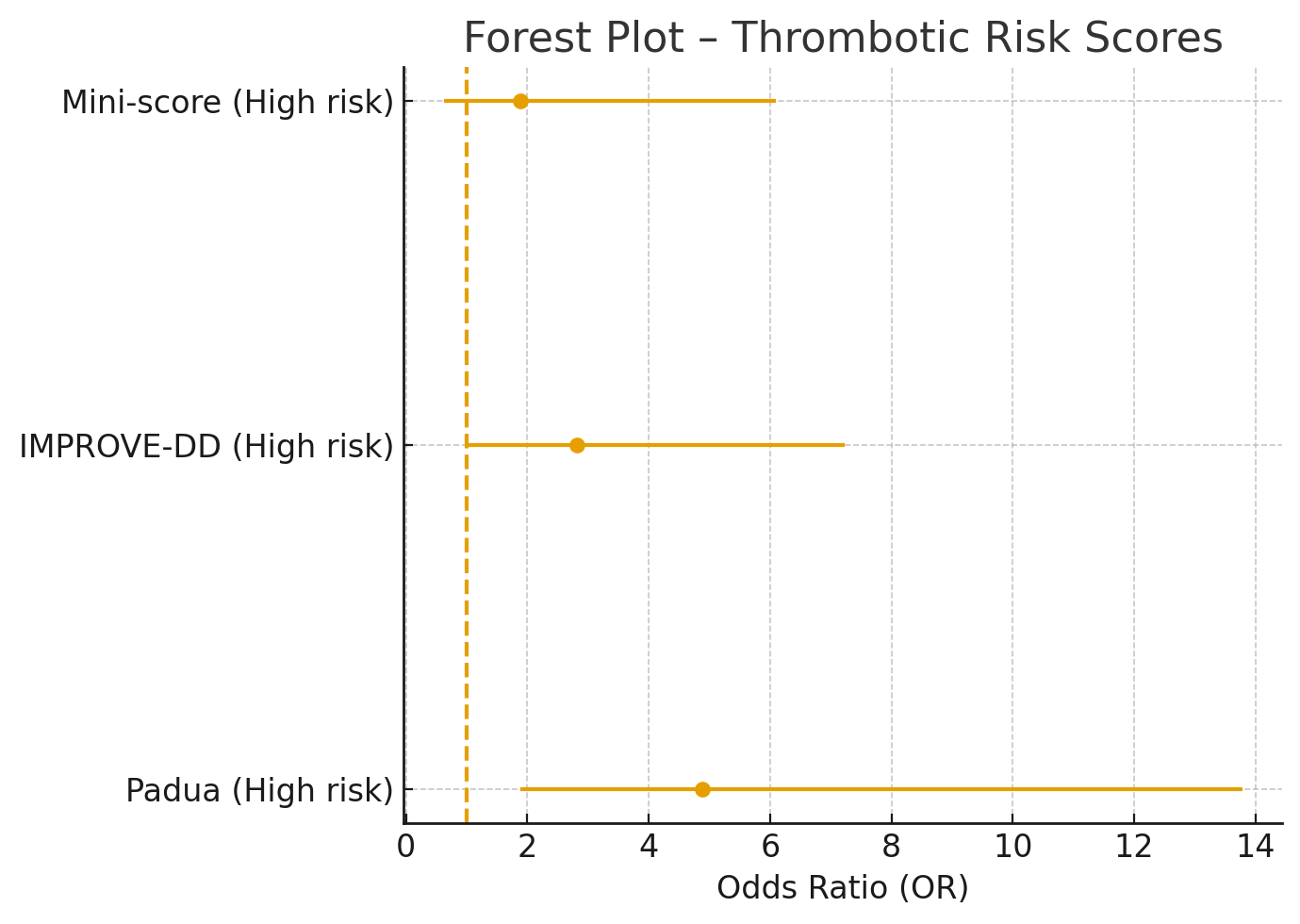

Supplement: Supplementary file 1 [file diagnostics-16-00724-s001.zip › Figure S6.png]
